# Murine Models of Acute Leukemia: Important Tools in Current Pediatric Leukemia Research

**DOI:** 10.3389/fonc.2014.00095

**Published:** 2014-05-07

**Authors:** Elad Jacoby, Christopher D. Chien, Terry J. Fry

**Affiliations:** ^1^Pediatric Oncology Branch, Center for Cancer Research, National Cancer Institute, National Institutes of Health, Bethesda, MD, USA

**Keywords:** acute lymphoblastic leukemia, xenografts, murine model, immunotherapy, adoptive, syngeneic

## Abstract

Leukemia remains the most common diagnosis in pediatric oncology and, despite dramatic progress in upfront therapy, is also the most common cause of cancer-related death in children. Much of the initial improvement in outcomes for acute lymphoblastic leukemia (ALL) was due to identification of cytotoxic agents that are active against leukemia followed by the recognition that combination of these cytotoxic agents and prolonged therapy are essential for cure. Recent data demonstrating lack of progress in patients for whom standard chemotherapy fails suggests that the ability to improve outcome for these children will not be dramatically impacted through more intensive or newer cytotoxic agents. Thus, much of the recent research focus has been in the area of improving our understanding of the genetics and the biology of leukemia. Although *in vitro* studies remain critical, given the complexity of a living system and the increasing recognition of the contribution of leukemia extrinsic factors such as the bone marrow microenvironment, *in vivo* models have provided important insights. The murine systems that are used can be broadly categorized into syngeneic models in which a murine leukemia can be studied in immunologically intact hosts and xenograft models where human leukemias are studied in highly immunocompromised murine hosts. Both of these systems have limitations such that neither can be used exclusively to study all aspects of leukemia biology and therapeutics for humans. This review will describe the various ALL model systems that have been developed as well as discuss the advantages and disadvantages inherent to these systems that make each particularly suitable for specific types of studies.

## Syngeneic Mouse Models of ALL

The identification of genetic changes in acute lymphoblastic leukemia (ALL) such as chromosomal translocations, gene amplification, and gene inactivation/deletion has revolutionized our understanding of the potential drivers of leukemogenesis. These have served as a basis for development of murine models that recapitulate human ALL. Genetic modification of ALL associated genes in primary cells with subsequent transplantation into mice, generation of transgenic mice that modify ALL associated genes in the lymphoid compartment, and chemical carcinogen induced mouse models have all been employed to try to model human pediatric ALL. In this section, we will focus on syngeneic models that have been shown to develop ALL that employ the first two methodologies as they utilize reverse genetics, which is more relevant to what occurs in human ALL.

## B Cell ALL Models

### *ETV6*-*RUNX1* *(TEL-AML1)*

The t(12;21)(p13;q22) chromosomal translocation encodes a novel fusion protein ETS variant 6 (ETV6)-runt-related transcription factor 1 (RUNX1) and is the most common rearrangement that occurs in ~25% of cases of pediatric B cell ALL (1). The ETV6-RUNX1 fusion protein consists of the basic helix loop helix (bHLH) domain of ETV6 combined with the transactivation domains of RUNX1. ETV6 belongs to the ETS family of transcription factors and has been found to have at least 20 different gene fusion partners and is also found to have other genetic alterations including deletions, point mutations, and promoter alterations in various leukemias (2). RUNX1 binds to DNA as part of the RUNX1/CBFβ transcription factor complex and the RUNX1 gene locus is commonly amplified in childhood ALL (3). Clinically, ETV6-RUNX1 expression is correlated with good prognosis (4).

Different groups have taken either bone marrow (BM) or fetal liver cells from mice and retrovirally transduced these cells to express ETV6-RUNX1 protein in an attempt to model human ALL. In three models, no incidence of leukemia was observed but increased numbers of precursor B cells were found with deficiencies of more mature B cells (5–7). In the BM models, increased ckit positive multi-potential progenitors were found with increased self-renewal potential of cells that expressed ETV6-RUNX1. In a fourth model of transduced BM, 2 of 9 mice that received translocation expressing BM developed ALL compared to 0 out of 20 control mice (8). Of the mice that had leukemia, one developed a T lineage ALL while the other developed a precursor B ALL (B ALL will be used to denote precursor B ALL throughout the rest of the review and a more mature B cell disease will be noted as such). In this same report, BM from p16^INK4a^/p19^ARF^ mice were also transduced with ETV6-RUNX1 and six of eight mice developed leukemia while no control mice developed leukemia.

Transgenic mice have also been employed to try to model ETV6-RUNX1 leukemias. The first study utilized the immunoglobulin heavy chain enhancer (Eμ) to drive expression of ETV6-RUNX1 in mice, however no leukemia was observed (9). This result was corroborated in subsequent reports where no leukemia was observed despite the use of differing gene promoters to express the translocation. Pre-leukemic changes in the BM were shown in a second transgenic model as pre-pro-B cell numbers were increased in mice (10). Based on three other reports, it appears likely that a second genetic event is necessary for the development of leukemia as co-expression of other genes and mutations induced by irradiation or carcinogens increased the incidence of leukemia (11–13). These data are supported by the clinical observation that ETV6-RUNX1 has been detected at birth prior to the development of leukemia (14).

### *E2A*-*PBX1*

The fusion protein consisting of the N-terminal transactivation domain of E2A protein and C terminal DNA binding homeobox domain of pre-B cell leukemia homeobox (PBX1) is expressed from the t(1;19)(q23;p13) and is detectable in 6% of childhood B ALL (15). E2A (transcription factor 3, TCF3) is a bHLH protein that codes for alternatively spliced transcripts for E12 and E47 proteins found to be required for B cell development (16). PBX1 was found to be necessary for lymphoid precursor development (17). Two groups have studied which domains are required for E2A-PBX1 transforming activity and found that the homeodomain of PBX1 which is responsible for binding to DNA is not necessary for oncogenesis (18, 19). However, the homeobox domain was important for blocking differentiation of myeloblasts (18). It was later found that a HOX cooperativity motif in PBX1 was needed to mediate transformation with E2A-PBX1 (20).

Initial attempts at modeling E2A-PBX1 driven leukemias were successful at causing myeloid leukemias and lymphomas, but not lymphoblastic leukemias. BM transduced with E2A-PBX1 from BALB/C mice led to the formation of acute myelogenous leukemia with an increase in immature blast cells in the BM (21). FVB transgenic mice in which E2A-PBX1 expression was driven with IgH V-gene promoter fused to Eμ died within 5 months of age of T cell lymphomas (22). These mice showed reduced numbers of T and B cell progenitors that were at 20% of normal levels. Though important to study the oncogenic potential of E2A-PBX1, these models did not reproduce the association of E2A-PBX1 expression seen in B ALL so the relevance of these model systems to human disease is not clear.

A later model succeeded in replicating E2A-PBX1 B ALL in mice (23). In this transgenic mouse, E2A-PBX1 is expressed under control of lymphoid specific Lck upstream sequence, Eμ enhancer, and TCR Vβ promoter. Interestingly, mice expressing the transgene that died at an earlier age were prone to developing T ALL in contrast to mice who developed leukemia at later timepoints, which succumbed to B ALL or mixed lineage ALL. E2A-PBX1 transgenic mice were crossed into a CD3ε^−/−^ background, which led to 40% of mice acquiring B ALL. These mice had a long latency time before symptoms of disease were observed and this latency was reduced when combined with Hox gene overexpression by viral insertional mutagenesis. In addition to *Hox* genes, *Pim1, Notch1*, and downregulation of *INK4A-ARF* by *Bmi-1* have also been shown to cooperate with *E2A-PBX1* in oncogenesis suggesting the importance of secondary hits in the development of *E2A-PBX1* driven leukemias (24–26).

### *E2A-HLF* t(17;19)(q22;p13)

E2A-hepatic leukemia factor (HLF) protein is the result of t(17;19)(q22;p13) translocation most commonly found in adolescents as a high risk pro-B cell ALL, yet is a rare event (27). Similar to the *E2A-PBX* gene fusion, the N-terminal transactivation domains of E2A are joined to the basic leucine zipper dimerization domain of HLF. HLF is a member of the proline and acidic amino acid-rich basic leucine zipper (PAR bZip) transcription factor family and mice deficient in this protein develop without any cancer pathology (28). Both transactivation domains of E2A and the basic leucine zipper dimerization domain of HLF were required for transformation of NIH3T3 mouse fibroblasts (29). The transforming ability may in part be due to upregulation of LIM domain only 2 (*LMO2*) (30).

Two transgenic mouse models of *E2A-HLF* were developed with slightly different results. The first model used the Eμ enhancer with the SV40 promoter to drive expression of the translocation (31). In this report, they described changes in the precursor T cell populations in the thymus and spleen with mature splenic B cells expressing low levels of *E2A-HLF*. Sixty percent of mice became sick with lymphomas, 90% of which were derived from a T cell lineage, and the remaining 10% of B cell origin. A second model also used the Eμ enhancer with its corresponding promoter to express *E2A-HLF* (32). An increase in T cell apoptosis in the thymus and a block in the maturation of B cells in the spleen was observed. These mice showed incomplete penetrance with 5 of 26 mice developing T ALL.

A later study used BM transduction to study the leukemogenic inducing potential of *E2A-HLF* (33). BM was transduced with a murine stem cell retrovirus to express transgenes and grown *in vitro* on irradiated AC-6.21 stromal cells before transplantation. These transduced cells without additional mutations did not induce leukemia in non-irradiated hosts. However, BM cells that were transduced to express *E2A-HLF* with *Bcl-2* and transplanted immediately into lethally irradiated hosts did develop B ALL, which is similar to the disease seen in human B ALL.

### *BCR*-*ABL*

Breakpoint cluster region (BCR)-ABL is the protein product caused by the t(9;22)(q34;q11) translocation (also known as the Philadelphia chromosome) is most commonly found in CML, but also accounts for 20–40% of adult B ALL and 3–5% of childhood B ALL cases (34, 35). Expression of the translocation product is controlled by the *BCR* promoter, which is ubiquitously expressed throughout the body (36). *BCR* null mice show alterations in activity of neutrophils (37) while *ABL1* knockout mice exhibit wasting of the spleen and thymus as well as significant loss of B and T cell numbers (38, 39). Two versions of the fusion protein associated with ALL are generated from different breakpoints leading to a 210-kDa protein (p210) and a 190-kDa protein (p190). The p210 isoform is detectable in 33% of adult Philadelphia chromosome positive (Ph^+^) ALL with the rest of cases expressing the shorter isoform. The p190 accounts for 90% of Ph^+^ ALL cases in childhood ALL. The BCR-ABL fusion protein is a constitutively active non-receptor tyrosine kinase due to removal of the domains of ABL1 that normally keep ABL1 in an inactive closed conformation (40) and this deregulation leads to transformation. There have been many reports to study the function of the *BCR-ABL* translocation in many leukemia models, however this section will focus on the work that pertains to studies of ALL in *BCR-ABL* model systems.

Early attempts to model ALL in transgenic mice used the metallothionein-1 promoter, which has broad tissue expression in the mouse, to express p190 *BCR-ABL* (41). This promoter was chosen as attempts to use the *BCR* promoter to drive expression of *BCR-ABL*, as occurs in human leukemia, led to embryonic lethality of mice. Of ten mice examined, two mice developed AML while six mice were diagnosed with B ALL. Though Heisterkamp et al. showed embryonic lethality using a *BCR* promoter, another report showed that a p190 *BCR-ABL* knock-in model, which has expression of p190 from the endogenous *BCR* gene locus, develops B ALL with high frequency in chimeric animals (42). In contrast, expression of p210 from the endogenous *BCR* locus does not result in neoplastic transformation of hematopoietic cells to form leukemia (43). Further work from the Heisterkamp group explored what happens when the p210 isoform of *BCR-ABL* is expressed using the metallothionein-1 promoter (44). p210 transgenic mice became sick from B, T, and myeloid leukemia with a long latency period while mice that expressed p190 only developed B ALL with a rapid disease course. A second (Honda et al.) publication also described a p210 *BCR-ABL* model driven by the same metallothionein-1 promoter (45). Interestingly, in this model two of six mice developed leukemia committed to the T cell lineage. The reasons underlying the difference in the spectrum of diseases seen in these p210 models are not clear, though differences in either strains of mice used or differences in promoter sequences used may contribute. Honda et al. created another *BCR-ABL* transgenic mouse that used the mouse *Tec* promoter to regulate p210 *BCR-ABL* expression (46). Tec is a cytoplasmic protein tyrosine kinase that is expressed in hematopoietic progenitor cells. Two of five founder mice developed ALL and the progeny of one of these mice developed CML at 1 year of age with some mice showing hallmarks of blast crisis in the lung. Another group took advantage of advances in tetracycline transactivator (tTA) proteins to regulate gene expression of p210 *BCR-ABL* (47). They used a “tet-off” system with the mouse mammary tumor virus long terminal repeat (MMTV-LTR) promoter, which controlled tTA protein. This allowed expression of p210 in the absence of tetracycline in the water of mice and repression of expression when mice were given tetracycline. In this model, mice developed B ALL and initiation and maintenance of ALL was dependent on p210 expression.

Bone marrow transduction studies have shown that *BCR-ABL* is capable of inducing multiple types of leukemia. Although the initial intention of this study was to model CML, expression of p210 via a myeloproliferative sarcoma virus (MPSV) in BM cells from BALB/c led not only to CML, but B and T ALL and macrophage derived tumor in 13 of 30 mice studied (48). A follow-up report using the same p210 BCR-ABL protein (b3a2) in BALB/c BM transduction/transplant studies, compared another p210 BCR-ABL protein (b2a2), resulting from an alternative breakpoint, when driven by a murine stem cell virus (MSCV) U3 promoter found that both gave rise to B ALL without any evidence of myeloid leukemia (49). Yet another group utilized a MPSV system to express p210 *BCR-ABL* in mouse BM and also observed a broad range of diseases in two different mouse strains (50). In both DBA/2 and C57BL/6 mice, macrophage and erythroid derived tumors were found, while in lymphoid derived tissues DBA/2 mice developed B cell lineage lymphomas while C57BL/6 mice developed T lineage lymphomas. In contrast to the previous report classical CML was not observed in either of these strains of mice. Another BALB/c BM transduction model was used with a Moloney murine leukemia virus (Mo-MuLV) to express p210 *BCR-ABL* with half of the animals developing CML like disease while the other half developed pre-B cell lymphomas (51). Another study using BALB/c mice used BM transduced with MSCV controlling expression of p190, p210, or p230 *BCR-ABL*, which is commonly associated with chronic neutrophilic leukemia (52). When BM from mice pre-treated with 5-fluoro uracil (5-FU), which enriches multi-potential myeloid cells in the BM, were transplanted to recipient mice all three isoforms of *BCR-ABL* gave rise to CML like disease in all mice with similar latency for development of disease. When the BM without 5-FU treatment was used for transduction and transplant, mice developed diseases of various lineages, which included not only CML like but also B ALL and macrophage lineage disease. The p190 isoform in the latter experiment showed more aggressiveness as these mice acquired disease at earlier timepoints with an increased incidence of B ALL. BM185 cells are another model system in which BALB/c BM is grown in early B cell progenitor Whitlock Witte cultures and transduced with p190 *BCR-ABL*. These cells when intravenously transplanted into mice developed B ALL with as little as 1000 cells in 3 weeks with 100% penetrance. (53). These findings underscore the complexity of modeling human disease as expression of the same cancer associated protein can have vastly different effects in mice and may be due to strain differences or use of alternative viral vectors. In addition, the cell of origin is another potential factor in disease variability highlighted by the fact that transplantation of a late onset CML like disease driven by p210 *BCR-ABL* in a BM transduction/transplant model led to not only myeloid disease, but also B and T ALL (54).

### *MLL* fusions

The mixed lineage leukemia (*MLL*) gene is most frequently found rearranged in infant ALL accounting for two-thirds of infant ALL cases and less frequently in AML and confers a worse prognosis (55, 56). There have been 121 rearrangements of *MLL* identified with 79 translocation partner genes characterized molecularly (57). The seven most common rearrangements involve *AF4, AF9, ENL, AF10, ELL, AF6*, or derive from partial tandem duplications of the *MLL* gene (*MLL*-PTD). The *MLL*-PTD are rare in acute lymphoblastic leukemia (58) and have not been modeled thus far and will not be covered in this review. Despite the rare instances of ALL in these models, we will highlight the work that has been done in mice with these common rearrangements that have been identified in ALL.

#### *MLL*-*AF4*

*MLL-AF4/AFF1* is the product of t(4;11)(q21;q23) and account for about 50% of cases of MLL rearrangement. Mouse models have used various approaches to express the MLL-AF4 protein. A standard transgenic mouse using pronuclear injection of MSCV *MLL-AF4* had B cell neoplasms found in the spleen, liver, lung, and blood after a long period of latency (59). Knock-in mouse models were also generated by using the *MLL* gene locus to express *MLL-AF4*. The first model used traditional gene targeting to fuse human *AF4* to exon 7 of mouse *MLL* (60). These mice developed lymphoid and myeloid hyperplasia with the most common malignancies of B cell lineage at 22 months of age. The second knock-in mouse was an inverter mouse that utilized a Cre/Lox system to cause a recombination event to happen after Cre expression that would cause *MLL-AF4* to be expressed instead of normal *MLL* (61). These mice developed more mature B cell neoplasms as opposed to B ALL from which the cDNA for *AF4* was originally isolated to generate the mice. The last model made use of a Lox Stop Lox cassette to halt transcription of *AF4* in the *MLL* locus prior to Cre recombination (62). *MLL-AF4* was only expressed in cells in which Cre recombination occurred and led to ALL and AML in mice.

Bone marrow transduction has also been used to model *MLL-AF4* leukemogenesis. In the Lox Stop Lox model, BM transduced with a transiently expressed Cre led to ALL, AML and MLL after transplant back into mice (62). In a separate report using MSCV *MLL-AF4* transduced BM, leukemogenesis required expression of the normal *MLL* gene (63). Another study found that *MLL-AF4* and its reciprocal translocation *AF4-MLL* were both able to cause ALL of all subtypes (B, T, mixed lineage) in mice that were transplanted with BM that expressed each alone or when co-expressed (64).

#### *MLL*-*AF9*

The t(9;11)(p22;q23) chromosomal rearrangement produces the MLL-AF9/MLLT3 leukemia associated protein. Two mouse models have been created to model *MLL-AF9* leukemia. *MLL-AF9* was targeted to the MLL locus in mice and expressed using native *MLL* gene regulatory elements (65). These mice only developed AML. In the second model, the investigators took a novel approach using Cre recombination where single LoxP sites were introduced into the endogenous *MLL* and *AF9* genes to create Cre mediated interchromosomal recombination of *MLL* and *AF9* (66). Chimeric mice generated using this strategy developed acute leukemias and in mice analyzed and was classified as AML. BM transduction/transplantation (tp) model AML has been recently reported (67). *MLL-AF9* has also been transduced in granulocyte macrophage progenitors to produce AML (68).

#### *MLL*-*ENL*

*MLL-ENL* is the product of the t(11;19) (q23;p13.3) translocation. The only reported interchromosomal gene recombined mouse model of *MLL-ENL* developed myeloid leukemias (69). BM transduction models have been made but not all studies have involved transplant into mice. MSCV *MLL-ENL* was introduced into early hematopoietic progenitors and transplanted into mice, which led to myeloid leukemias (70). In a separate study, MSCV *MLL-ENL* transduced BM grown in *ex vivo* conditions favoring lymphopoiesis without a stromal feeder layer induced a continuously growing cell population (71). The proliferating cells were initially B220^+^CD19^+^, but after continual growth a B220^+^CD19^−^ population dominated the culture. The B220^+^CD19^−^ cells could induce splenomegaly and infiltrated the thymus of mice and despite being morphologically myeloid, they retained B220 positivity showing a mixed lineage phenotype. Later BM transduction models used conditional *MLL-ENL*. A mutated estrogen receptor fusion protein of MLL-ENL was used, but not introduced into animals so the resultant disease was not reported (72). A tetracycline regulatable *MLL-ENL* when used to transduce BM was able to induce AML and expression of MLL-ENL protein was required for leukemic growth even in leukemias that acquired secondary mutations (73).

#### *MLL*-*AF10*

*MLL-AF10* is derived from the rearrangement t(10;11)(p13–15;q14–21). Only BM transduction studies have been done using this gene fusion. Two studies use the same MSCV based BM transduction with *MLL-AF10* and both models develop myeloid lineage disease (74, 75). An additional model studied the cooperativity of *MLL-AF10* with the activating mutation Kras G12C (76).

#### *MLL*-*ELL*

The only published study of *MLL-ELL* gene fusions utilized a MSCV based BM transduction strategy, where C terminal domain of ELL was shown to be required for transformation of myeloid progenitor cells. In this study, there was no data shown for transplantation of these transduced cells into mice but *MLL-ELL* expression caused immortalization of myeloid progenitors *in vitro* (77).

#### *MLL*-*AF6*

The only MLL-AF6 model of leukemia used BM transduction to demonstrate the requirement of histone methyltransferase Dot1l for MLL-AF6 induced leukemogenesis (78).

### PAX 5 deletions

Somatic mutations and deletions of PAX5 have been found in about 30% of pediatric B ALL samples (79). To date, only haploinsufficiency of PAX5 was shown to be important for induction of leukemia in an activated STAT5 B ALL mouse model (80).

### *IGH*-*Myc*

Myc translocations occur in about 5% of both adult and childhood ALL (81). Transgenic mice that express Myc under control of IGH enhancers showed B cell lymphomas with leukemic involvement at later timepoints (82, 83). Knock-in of Myc into the native IGH locus induced B cell neoplasms (84). BM transduction studies using p53 null BM transduced with Myc led to B cell lymphomas (85).

## T Cell ALL

### NOTCH1 activation

NOTCH1 is a type I transmembrane receptor that is involved in signal transduction and is the only *NOTCH* gene involved in T ALL (86). Activation of the NOTCH1 pathway is found in over 50% of T ALL cases (87). In concordance with this clinical data, NOTCH1 mutation has been identified in two transgenic mouse T ALL models induced by KRas G12D (88, 89). No T ALL transgenic models have been shown to be solely driven by *NOTCH1* mutations, but BM transduction/transplantation studies have shown that NOTCH1 activating mutants can drive T ALL formation. A L1601PΔP mutant, which has a L1601P and frameshift mutation in the PEST domain of NOTCH1 was shown to inefficiently induce T ALL in mice after transduction and transplantation into mice (90). A separate NOTCH1 mutant (ICN1) which consists of the transmembrane and intracellular domain of NOTCH1 was able to induce T ALL in BM cells transduced with ICN1 after transplant into mice (91). Transgenic mice expressing ICN1 in T cell progenitors have also been shown to develop T ALL (92).

### Inactivation SCF^FBW7^

FBW7 is subunit of the SCF-type E3 ubiquitin ligases. *FBW7* mutations are found in many human cancers such as T ALL, which corroborates its role as a tumor suppressor (93). Conditional inactivation of *FBW7* in T cell lineage cells in mice caused thymic hyperplasia likely due to Myc accumulation (94).

### TAL1 and LYL1 mutations

TAL1 is a class II bHLH transcription factor that is a master regulator for commitment to the hematopoietic lineage. *TAL1* mutations or translocations are found in about 25% of childhood T ALL (95, 96). *Lck* promoter driven *TAL1* transgenic mice also develop lymphomas of a predominantly mixed T and B cell phenotype (97). Transgenic mice where a *TAL1* DNA binding mutant is expressed still develop T cell leukemia/lymphoma (98). bHLH proteins are commonly found to interact with other bHLH proteins as heterodimers to regulate gene expression and a potential hypothesis for the role of TAL1 in T ALL is that overexpression of TAL1 and its mutants disrupt the normal gene program by interacting with other transcription factors that regulate T cell development such as E2A proteins (98).

LYL1 is also a class II bHLH transcription factor that heterodimerizes with class I bHLH transcription factors. Transgenic mice that express *LYL1* developed T cell lymphomas positive for both CD4 and CD8 and mature B cell lymphomas (99). LYL1 is also proposed to alter transcription through its interaction with other transcription factors such as E2A (99).

### *LIM* *domain* *only* *2*

LIM domain only 2 is a cysteine rich protein that cooperates with other nuclear proteins to regulate gene transcription. Three transgenic models of LMO2 have been studied in relation to T ALL and differ in choice of promoter to drive expression of LMO2. Metallothionein-1 promoter led to 10% of LMO2 transgenic mice developing CD4/CD8 double positive, single positive, and double negative T cell neoplasms (100). Higher penetrance was seen in 75% of mice that expressed LMO2 under control of the CD2 promoter (101). A third mouse reported used *Lck* promoter to express LMO2 and showed cooperation with TAL1, a known binding partner to LMO2, to induce T ALL (102).

### *CALM*-*AF10*

The translocation t(10;11)(p13;q14–21) creates the CALM-AF10 fusion protein. They are most commonly found in TCRγδ expressing T ALL. *Vav* driven *CALM-AF10* in a transgenic mouse model led to mixed lineage ALL (103). BM transduced with MSCV driven CALM-AF10 and transplanted into mice led to leukemias that expressed predominantly myeloid markers Mac1 and Gr1 and more rarely B cell marker B220 alone or co-expressed with Mac1 and Gr1 (104). A recent study also showed that CALM-AF10 transformation is dependent on the histone methyltransferase Dot1l (105).

### Summary of syngeneic mouse models of ALL

There has been significant progress made in trying to model ALL in mice. The number of exogenous and endogenous promoters to control expression of leukemia associated genes and mutations has grown significantly and their tissue expression has been well characterized. Advances in conditional mouse technology have also been rapidly incorporated into various models with inducible models providing exquisite control of transgene expression in cell lineages. Through the use of BM transduction, a more rapid disease course is possible as secondary mutations can be introduced that cooperate with primary mutations. This is important because in the majority of ALL models long periods of latency are seen with expression of single leukemia associated aberrations in mice, which make study of these lesions difficult and time consuming. The development of these ALL models (summarized in Table 1) has contributed greatly to our understanding not only of pathways that underlie leukemogenesis, but also helps us identify potential therapeutic targets in these pathways.

**Table 1:** **Syngeneic mouse models of ALL**.

Gene alteration	Murine model	Diseases observed	Secondary mutations	Reference
*ETV6-RUNX1*; t(12;21)(p13;q22)	TM	B ALL	*Cdkn2a*^−/−^, chemical carcinogen induced, insertional mutagenesis	(11–13)
	BM	T ALL, B ALL	Not reported	(8)
*E2A-PBX1*; t(1;19)(q23;p13)	TM	T, B, and mixed lineage ALL	*Pim1, Notch1*, and downregulation of *INK4A-ARF* by *Bmi-1*	(23–26)
*E2A-HLF*; t(17;19)(q22;p13)	TM	T ALL	Not reported	(32)
	BM	B ALL	*Bcl-2*	(33)
*BCR-ABL*; t(9;22)(q34;q11)	TM	AML, T ALL, B ALL	Not reported	(41, 42, 44–47)
	BM	CML, T ALL, B ALL, macrophage derived tumor	Not reported	(48, 49, 52–54)
*MLL-AF4*; t(4;11)(q21;q23)	TM	B lymphoma, B ALL, AML	Not reported	[60; 62]
	BM	B ALL, mixed lineage ALL, AML	Not reported	(62, 64)
*MLL-ENL*; t(9;11)(p22;q23)	BM	B ALL, mixed lineage ALL	Not reported	(71)
*Pax5* haploinsufficiency	TM	B ALL	STAT5 activation	(80)
*IGH-Myc*	TM	B lymphoma, B ALL	Not reported	(82, 83)
*NOTCH1* activation	TM	T ALL	KRas G12D	(88, 89, 92)
	BM	T ALL	Not reported	(90, 91)
*TAL1* mutations	TM	T lymphoma, T ALL	Not reported	(98)
*LMO2*	TM	T lymphoma, T ALL	Not reported	(100–102)

*TM, transgenic mouse model; BM, bone marrow transduction/transplantation model; T ALL, T cell acute lymphoblastic leukemia; B ALL, B cell acute lymphoblastic leukemia*.

Despite the promise in these models, there seems to be some discrepancies in the disease observed driven by these ALL associated genetic events. In trying to study ALL, expression of ALL associated genes/mutations/translocations has led to AML in a significant number of instances. This could be due to promoter choice, cell of origin or cross species protein expression. Various promoters have been used to try to express leukemia associated alterations in genes and promoter choice affects the cells that express those gene products. This would explain why the same leukemia factor expressed under control of alternative gene promoters leads to different diseases in mice than in humans. Additionally, there may be complications that arise in expressing human leukemia associated proteins in a mouse. Protein–protein interactions that exist in humans may be absent or different in mice and therefore proper reproduction of human disease in mice may become more difficult.

There are advantages for using syngeneic models of ALL. The contribution of the microenvironment to the development of many cancers is an important topic of study in carcinogenesis. Cytokines, growth factors, and cell–cell interactions provided by the local tumor microenvironment influence how cancers initiate and develop. Syngeneic models of ALL can be utilized to study the tumor microenvironment and its contribution to leukemogenesis. There are factors that contribute to leukemogenesis that do not signal across species and therefore would be important to study in a single species model. For example, the role of thymic stromal lymphopoietin receptor overexpression (TSLPR/CRLF2) in B ALL is being studied. TSLP, the ligand for TSLPR, does not signal cross species so study of TSLPR overexpressing leukemia must be pursued in a single species model. Syngeneic models of TSLPR overexpressing B ALL would allow study of disease course in the presence of ligand at physiologic doses that is not possible in a xenograft model. Expression of human TSLP in mice would be required to study human TSLPR overexpressing B ALL in xenograft model systems where mimicking physiologic levels of the ligand could be problematic.

## Xenograft Models: Development of the Human ALL-Bearing Mouse

The pursuit of an animal model that would provide *in vivo* insights of direct relevance to human ALL has been undertaken by many groups using multiple types of immunodeficient mice to allow engraftment of human cells. The first candidate was the athymic nude mouse, developed in the late 1960s. Lack of T cells allowed engraftment of some human tissues and tumors, but intact NK cells and B cells prevented engraftment of human hematopoietic cells. Still, by conditioning mice with radiation, initial studies demonstrated some ability for leukemia cells to engraft (106–108). Nonetheless, engraftment of cells administered intravenously was extremely difficult. Thus, in these models, recipients were inoculated with leukemia cell lines in the intradermal, subcutaneous or intraperitoneal compartments, and at times required an addition of irradiated cells from a fibrosarcoma cell line in order to engraft (107). The results were ascites (106) or solid masses (108) of leukemia origin, which allowed studying of new drugs and therapeutic approaches. However, the leukemia lacked its “normal” BM niche and natural course in extramedullary organs. Thus, the study of homing, engraftment, persistence, course, and therapeutic approaches in the native leukemia niche was impossible in this model.

Introduction of the severe combined immunodeficiency (SCID) mouse on a CB17 background (109), was a major advance in the field, allowing marrow engraftment of human hematopoietic cells [reviewed by Shultz (110)]. These mice have a deletion in *Prkdc*, an essential protein involved in DNA repair following VDJ recombination in T and B cells. Hence, these mice lack adaptive immune cells, but retain NK cells as well as innate immunity; therefore, these models often required conditioning with sublethal irradiation to engraft human leukemia (111), though some groups overcame this need by repeated *in vivo* transfers of the leukemia cell line. An important breakthrough was the use of SCID mice to engraft patient derived primary leukemia cells following an IV injection (112). This resulted in engraftment and proliferation of the leukemia *in vivo*, with infiltration of the spleen, liver, kidney, lung, central nervous system, (112) lymph nodes and ovaries (113), thus imitating extramedullary involvement. Also, compared to nude mice, SCID mice engrafted leukemia following a lower inoculation dose (114).

Several attempts were made to genetically manipulate the SCID mouse to increase the engraftment ability of the SCID mouse. The SCID-beige mouse was developed by adding a mutation in the lysosome trafficking regulator (*Lyst*) to the *Prkdc^scid^* mutation, on a Balb/c or CB57/BL/6J background. This resulted in lack of T and B cells as well as neutropenia and reduced NK function; moreover, these strains avoided the late immune-reconstitution seen in SCID mice (115). This model can engraft leukemia and has been used by a number of groups in leukemia research (116).

The NOD-SCID mouse was generated by crossing the SCID mouse (*Prkdc^scid^*) to the non-obese diabetic (NOD/Lt) mouse background, resulting in reduced NK function as well as impaired innate immunity derived from the NOD mutation in a diabetes-free strain (110, 117). This mouse showed increased rate of engraftment of human hematopoietic cells in general, including ALL cell lines and patient samples (118, 119). Moreover, impaired NK function reduced the need for conditioning with radiation prior to leukemia administration, though conditioning with irradiation was still used by investigators (120, 121). This was of importance, since these mice have a tendency to develop spontaneous thymic lymphoma, and have generally a short life span [median survival of 257 days (117)]. Human leukemia seemed to have stable properties when passed to an NOD-SCID mouse, with stable antigenic markers, T- or B-cell receptor rearrangements, karyotype, as well as a stable rate of engraftment in subsequent passes of the leukemia (121, 122).

Crossing the common-gamma-chain (interleukin 2 receptor gamma) knockout to the NOD-SCID mouse generated the NOD/SCID/γC^−^ (or NSG mouse), with multiple defects in both the innate and the adaptive immune system. Lack of NK cells omitted the need for irradiation prior to leukemia administration. NSG mice have superior engraftment of ALL cells in terms of time to engraftment (shorted by ~2 weeks compared with NOD-SCID mice), ability to detect blasts in the peripheral blood, and total number of cells required to engraft (123, 124). NSG mice can even be engrafted with as little as 1–100 cells derived from patients with poor-prognosis ALL (125). Again, as in the NOD-SCID model, patient derived B or T -lineage ALL engrafted in NSG mice have a stable phenotype and transcriptome, even after a several passes *in vivo* (126). The relative simplicity of engrafting human leukemia in these mice made the NSG model excellent for leukemia research.

In summary, the evolution of the immunocompromised mouse, with its ability to engraft human hematopoietic cells, provided *in vivo* models for studying human ALL. Nevertheless, there are some significant drawbacks in using these mice. Since they have profound immunodeficiency, they need to be housed in a protected pathogen-free environment. Moreover, they have an inherent tendency toward spontaneous lymphomas. Third, engraftment of human T cells (from BM transplant or immunotherapy experiments) results in high rate of lethal xenogeneic graft versus host disease (Xeno-GVHD).

Recently, inactivation of CD47 on a C57BL/6 mouse with Rag2 and Gamma-chain knockout (triple-knockout) allowed tolerance toward human hematopoietic stem cells. These triple-knockout mice had no GVHD at 29 weeks after transplantation, compared with one-third of NSG mice that develop GVHD at this point (127). This model has not been introduced to leukemia challenge, but it may be a promising avenue in the future, especially for immunotherapy models.

Utilizing these mouse strains (summarized in Table 2) to study leukemia require administration of leukemia cells, via intravenous route or direct into the marrow compartment (intratibial or intrafemoral) (128, 129). Commercially available cell lines or primary patient samples of ALL may be injected in xenogeneic mice, with different engraftment rates, depending on the mouse strain as well as the leukemia properties (discussed below).

**Table 2:** **Xenograft mouse strains used in human leukemia research**.

Strain	Mutation	Immune phenotype				Engraftment of human hematopoietic cells	Additional significant limitations	Reference
		T	B	NK	Innate	
Nude athymic		−	+	+	+	−	No IV engraftment	(106–108)
SCID	*Prkdc^scid^*	−	−	+	+	+	RS	(112, 113)
SCID-Bg	*Prkdc^scid^* *Lyst*	−	−	Low	±	+	RS, leak	(116)
NOD-SCID	*Prkdc^scid^* NOD/Lt	−	−	Low	±	++	RS, Th, leak	(119, 122)
NOD-SCID-gamma (NSG)	*Prkdc^scid^* NOD/Lt; I*l2rg^tm1Wjl^*	−	−	−	−	+++		(123, 125)
NOD-truncated-gamma (NOG)	*Prkdc^scid^* NOD/Shi; IL2rg^−/−^(truncated)	−	−	−	−	++		(159)

*RS, radiosensitivity; Th, tendency for thymic lymphomas; leak, leakiness phenomena of delayed reconstitution of some immune cells*.

## Questions Addressed in the Xenogeneic Mouse Model

### Microenvironment of ALL

The ability to engraft human leukemia in the murine BM provided an opportunity to study the leukemia niche within the marrow, as well as its progression and extramedullary involvement. Indeed, patient derived pre-B ALL cells that were intravenously administered to SCID mice caused multiple organ involvement, including hepatosplenomegaly, kidney and ovarian enlargement, as well as lymphadenopathy (112). Focusing on the BM niche is outside of this review’s scope, but some examples for utilizing the xenograft models in these studies can be given. For example, inhibition of CXCR4 in ALL samples administered to NOD-SCID mice showed increased sensitivity of the blasts compared with normal hematopoietic stem cells, pointing out the CXCR4–CXCL12 axis as significant in ALL homing (130). Messinger et al. searched for different adhesion molecules in ALL blasts in different organs of xenografts transplanted with primary patient samples of pre-B ALL. They identified VLA-4 and VLA-5 in the BM ALL blasts, but not in the liver or spleen, suggesting loss of these integrins may be a property of the leukemic cells prior to migration to extramedullary organs (131). CNS leukemia was studied initially in the SCID mouse. Eight days following IV administration of NALM-6 ALL cell line, PCR demonstrated evidence of CNS leukemia (132). This allowed experimental therapeutic tests of CNS leukemia.

### Leukemia stem cell

The study of leukemia stem cells or leukemia initiating cells (LIC) utilized xenograft models as well, mainly by sorting cells based on surface markers and assessing capability of initiating leukemia *in vivo*. Initial studies focused on the hematopoietic stem cell markers CD34 and CD133, as well as lineage specific markers (CD10, CD19, CD22, CD38 for B cells, CD3 and CD7 for T cells). Cox et al. sorted patient derived T ALL cells. They found that CD34^+^/CD4^−^ or CD34^+^/CD7^−^ cells were capable of engraftment in NOD-SCID mice, with self-renewal and potential for secondary and tertiary engraftments, marking these as LIC (121). CD34^+^/CD7^−^ cells had a higher engraftment rate in NSG mice compared with NOD/SCID mice (123), both resulting in engraftment, differentiation with similar immunophenotype to the original patient sample. In B-precursor ALL, CD34^+^CD19^−^ and CD34^+^CD10^−^ cells sorted from patients had long-term proliferation in NOD/SCID as well as NSG mice, compared with CD34^+^CD19^+^ cells, suggesting the prior sub-population to be LIC (123, 133). In contrary, other investigators have shown that CD34^+^CD19^+^CD38^±^ cells sorted from patients’ BM were able to initiate leukemia in NSG mice, had self-renewal properties, and resulted in lethal CD19^+^ leukemia in the host. Transplantation of patient derived CD34^+^CD38^−^CD10^−^CD19^−^ cells did not result in overt disease, suggesting the CD34^+^CD19^+^CD38^±^ as the LIC (134). When sorting primary patient samples on CD133, CD133^+^/CD19^−^ as well as CD133^+^/CD38^−^ cells engrafted at low inoculating doses (5.4–6.5 × 10^3^ cells), while CD34^+^CD19^+^ cells needed an almost 100-fold higher dose to engraft. Also, CD133^+^/CD19^−^ cells were resistant to vincristine and dexamethasone, suggesting this phenotype as chemo-resistant leukemia stem cells (135). Even more controversy was introduced when, via intrafemoral injections of purely sorted leukemia cells, le Viseur et al. showed that different populations of the leukemia have potential to re-establish leukemia in subsequent transplantations to NOD/SCID mice (128).

Studies of identical twins with leukemia suggested similar LIC with either *TEL-AML1* or *BCL-ABL* translocations (136, 137). To study whether the fusion proteins resulting from these translocations may generate a self-renewing pre-leukemic clone, human cord blood cells transduced with TEL/AML1 were transplanted into NOD/SCID mice, resulting in CD34^+^CD38^−/low^CD19^+^ population, similar to pre-leukemic phase seen in identical twins of the patients (129).

### Marker for aggressiveness/prognosis

Given the diverse nature of pediatric leukemia, a search for prognostic factors, which, at diagnosis, would allow a tailored therapy with escalation or de-escalation of treatment, is an ongoing mission. Several investigators attempted to use xenograft models to answer this question, by injecting primary leukemic cells or BM from leukemia patients into these mice.

Initial studies in SCID mice showed some correlation with prolonged remissions in patients whose cells did not engraft (111, 138, 139), but this was not consistent, nor was it consistently observed in the NOD-SCID mouse (120, 140). In a large cohort of patient derived pre-B ALL, only samples from low-risk patients showed correlation between engraftment rate (time to leukemia, TTL) in NOD-SCID mice and duration of remission (140, 141). Similar observations were made in the NSG mouse, with lower engraftment rate from patients who did not relapse (126). When assessing samples from relapsed patients, these tended to grow at a faster rate compared with samples from diagnosis when engrafted in SCID mice (111). The TTL or rate of engraftment of the relapsed sample in the NOD-SCID mouse correlated with remission duration (120). *In vivo* sensitivity to drugs, as assessed by survival of leukemia bearing mice following drug administration vs. saline, was also tested as a prognostic marker. Sensitivity to vincristine and dexamethasone, but not methotrexate, correlated with remission rate of patient derived ALL engrafted in NOD-SCID mice (120, 122). In general, these studies suggest that rate of engraftment in xenogeneic models may be used as a prognostic factor, but this method is complicated, expensive, and too difficult to be utilized in many centers.

### Therapeutic approaches

Most of the chemotherapy drugs used in treating pediatric ALL were developed prior to these *in vivo* models. However, in the past two decades, several novel therapies have utilized the xenograft model as an essential part of the pre-clinical evaluation. In an *in vitro* drug test, the target cells are continuously exposed to the tested compound. An *in vivo* model required scheduled doses, which have different pharmacodynamic and pharmacokinetic properties, similar to clinical administration of drugs (142). Treatment models use survival, or bioluminescence readings, as measures to evaluate *in vivo* treatment efficacy. This review is focused on the modeling system and will not comprehensively address all treatment approaches. Thus, we will discuss only selected therapies tested in the xenogeneic models, including chemotherapy, biologic agents, and immunotherapy.

New chemotherapy agents, as topotecan, have been evaluated in the SCID mouse. SCID mice had a long-term remission following topotecan administration. Also, the pharmacokinetics of topotecan in mice was similar to the pharmacokinetics in patients (143). mTOR inhibitors are also increasingly used in different pediatric tumors. Mouse models of ALL demonstrated that the mTOR inhibitor sirolimus was able to prolong survival of mice bearing ALL (144). Temsirolimus, another mTOR inhibitor, significantly prolonged survival of NOD-SCID mice injected with patient derived ALL and was found to be synergistic with methotrexate (145).

As previously mentioned, leukemic blasts require CXCR4 to stay in their BM niche. CXCR4 antagonists (Plerixafor or AMD11070), used for hematopoietic stem cell mobilization, were found to increase the proportion of actively cycling ALL cells in the peripheral blood of NOD-SCID mice, prolong survival of NSG mice, and increase either strain’s sensitivity to vincristine (130, 146).

Histone DeAcetylase (HDAC) inhibitors are also novel cancer medications. Balb/c-Rag^−/−^gammaC^−/−^ mice bearing patient derived T or B ALL were treated with LBH589, a HDAC inhibitor. This treatment resulted in prolonged survival of the mice compared with standard vincristine and dexamethasone treated mice, while combination of all three therapies prolonged survival even further (147). Other novel therapies based on specific leukemia biology, such as CBP/catenin inhibitor (ICG-001) (148), interleukin 27 administration (149), spleen-tyrosine kinase (SYK) inhibitors (150), anti-connective tissue growth factor (CTGF) monoclonal antibody (151) for pre-B ALL, and anti-Notch1 monoclonal antibodies or for T ALL (152), were all shown to prolong survival and/or reduce engraftment in NOD-SCID or NSG mice, with or without additional chemotherapy.

Ricin-conjugated immunotoxin were tested in SCID mice and shown to induce a prolonged remission compared with controls (114). Messinger et al. combined an anti CD19 immunotoxin conjugated to poke-weed antiviral protein with several chemotherapeutic agents, and showed that only combination with cytarabine led to prolonged survival in SCID mice (153). More recently, a combination of two ricin-conjugated monoclonal antibodies (against CD19 and CD22) was shown to be effective in combination with cytarabine against ALL-bearing NSG mice (154).

Use of adoptive-T cell based immunotherapy is an increasing focus of basic as well as clinical research. As these are cell-based therapies that proliferate *in vivo*, xenograft models were an essential part of describing these therapies, and of the pre-clinical evaluation. Cytotoxic T lymphocytes (CTLs) designed to target specific leukemia cells, resulted in remission of NOD/SCID mice bearing ALL (155). There is a growing interest in genetically engineered chimeric-antigen-receptor (CAR) T cells. Ability to track leukemic progression with luciferase-transduced leukemia cell lines was used to assess *in vivo* activity of CAR-T cells. A CD19-CAR administered to NOD/SCID mice inoculated with Daudi cells (156), and to SCID-Beige mice inoculated with NALM-6 cells (116), resulted in disease remission, as did a CD22 CAR in NSG mice inoculated with NALM-6 cells (157). These models can be used to assess treatment efficacy and treatment failures (escape mechanisms) on ALL cell lines as well as primary samples, and were able to identify the periodontal region as a new sanctuary site of leukemia, in which homing of the CAR-T cells was inadequate (116). Some major disadvantages of these models can also be elucidated when testing cell-based therapies: lack of a native immune system limits true studies of engraftment and persistence of adoptively transferred cells, in an environment without competition and/or native cytokine support. Also, there is an inherent limited survival of human T cells on some models (SCID-Beige mice) (116) and graft versus host disease frequently arises from the adoptively transferred human T cells against the mouse (158).

## Summary

As described in this review, there are multiple murine models in current ALL research. The xenogeneic model, especially the NOD-SCID and NSG strains, has superb ability to engraft human hematopoietic cells and human leukemia, and may serve as an appropriate platform for *in vivo* research of human-derived leukemia, utilizing either cell lines or patient derived cells. Given the diverse landscape of acute leukemia as far as genetic abnormalities, the ability to engraft patient derived cells is a major advantage of xenogeneic models. Also, they may serve as *in vivo* platforms for drug testing against ALL. Moreover, the relative simplicity of using bioluminescent imaging of leukemia (cell lines or patient samples) in xenogeneic mice for evaluation of disease has provided a method to detect proliferation and progression of disease without sacrificing multiple mice (144). Though bioluminescent imaging has been used in syngeneic models of other cancers, it has not been successfully utilized in leukemia.

Major limitations of these models include limited life span and even more – lack of a native immune system, which is crucial to this model’s engraftment properties. The immune system’s role in the biology of leukemia development and ALL niche cannot be assessed in these models. Also, novel therapies, as immunomodulatory drugs or bispecific T cell engagers (BiTE) antibodies, are based on the immune system and cannot be completely assessed in this model.

The syngeneic model allows the study of leukemia in an immunocompetent host. This model can be used extensively to study the leukemia microenvironment in different organs. Though these models are driven by common human ALL genetic rearrangements or oncogene overexpression, in mice (as in humans) these translocations are not sufficient for leukemogenesis. Thus, these mice have additional germline alterations, which may not represent the additional hits in human disease. Also, these are limited models that cannot match the full spectrum of human acute leukemias. Moreover, given that these leukemia models are of murine origin, many drugs developed for human leukemia cannot be tested given the species barrier.

In summary, murine models play an essential part of leukemia research as well as therapeutic development. The benefits and limitations of each model should be weighed when addressing a specific study question. Altogether, the development of both xenogeneic as well as syngeneic models create many opportunities to answer fundamental questions of leukemia biology, and serves as a platform for the development of novel therapies.

## Conflict of Interest Statement

The authors declare that the research was conducted in the absence of any commercial or financial relationships that could be construed as a potential conflict of interest. The content of this publication does not necessarily reflect the views of policies of the Department of Health and Human Services, nor does mention of trade names, commercial products, or organizations imply endorsement by the U.S. Government.
